# Entropy-Based Graph Clustering of PPI Networks for Predicting Overlapping Functional Modules of Proteins

**DOI:** 10.3390/e23101271

**Published:** 2021-09-28

**Authors:** Hoyeon Jeong, Yoonbee Kim, Yi-Sue Jung, Dae Ryong Kang, Young-Rae Cho

**Affiliations:** 1 Department of Biostatistics, Wonju College of Medicine, Yonsei University, Wonju-si 26426, Gangwon-do, Korea; hoyeonjeong@yonsei.ac.kr (H.J.); dr.kang@yonsei.ac.kr (D.R.K.); 2 National Health Big Data Clinical Research Institute, Wonju College of Medicine, Yonsei University, Wonju-si 26426, Gangwon-do, Korea; 3 Division of Software, Yonsei University Mirae Campus, Wonju-si 26493, Gangwon-do, Korea; yoonbee7@yonsei.ac.kr (Y.K.); yisue@yonsei.ac.kr (Y.-S.J.); 4 Division of Digital Healthcare, Yonsei University Mirae Campus, Wonju-si 26493, Gangwon-do, Korea

**Keywords:** protein–protein interaction networks, PPI networks, functional modules, protein complexes, graph clustering, graph entropy, overlapping community detection

## Abstract

Functional modules can be predicted using genome-wide protein–protein interactions (PPIs) from a systematic perspective. Various graph clustering algorithms have been applied to PPI networks for this task. In particular, the detection of overlapping clusters is necessary because a protein is involved in multiple functions under different conditions. graph entropy (GE) is a novel metric to assess the quality of clusters in a large, complex network. In this study, the unweighted and weighted GE algorithm is evaluated to prove the validity of predicting function modules. To measure clustering accuracy, the clustering results are compared to protein complexes and Gene Ontology (GO) annotations as references. We demonstrate that the GE algorithm is more accurate in overlapping clusters than the other competitive methods. Moreover, we confirm the biological feasibility of the proteins that occur most frequently in the set of identified clusters. Finally, novel proteins for the additional annotation of GO terms are revealed.

## 1. Introduction

A functional module is a separable entity in which the functions can be separated. Functional modules overlap with each other because a protein performs multiple functions under different conditions [1]. A protein complex is a multiprotein unit composed of several proteins linked by non-covalent bonds. A protein can be included as a subunit in multiple complexes of oligomeric structures. Functional modules or protein complexes can be predicted using protein–protein interactions (PPIs) from a systematic perspective. PPIs can be represented as a network, which is an undirected graph. The discovery of the entire set of functional modules from genome-wide PPI networks is an important goal of functional genomics [2]. Detecting overlapping clusters is also useful for predicting functional modules at the genome scale [3].

Various graph clustering algorithms have been applied to biological networks. Graph clustering algorithms can be divided into two groups: partition-based and local search algorithms. Partition-based algorithms search for the optimal partitioning of a graph. For example, Markov clustering (MCL) [4] is a partition-based clustering algorithm for weighted networks. This algorithm strengthens and weakens the connections iteratively through Markov chains to determine the optimal partition. InfoMap [5,6] is also a partition-based clustering algorithm that was originally designed for directed and weighted networks. However, it can be applied to undirected graphs by considering all the edges as bidirectional. InfoMap uses an entropy metric to determine the optimal partition by minimizing both the local entropy per cluster and the global entropy. Hierarchical algorithms can be included in the category of partition-based clustering. They repeatedly merge the closest subgraphs or recursively divide a graph into two subgraphs to achieve the best partition. The major characteristic of these partition-based clustering algorithms is that they are unable to detect overlapping clusters, that is, two or more clusters do not share any nodes.

Local search algorithms repeatedly search for the best cluster in a local area to generate a set of clusters. They use their own modularity functions for the local optimization. MCODE [7] is one of the most prevalent graph clustering algorithms for biological networks. This algorithm follows the seed growth procedure for a local search. For each cluster, the selected seed node grows by adding neighbors that have a score above a given threshold. As a severe disadvantage, MCODE requires the setting of many parameters for scoring and adjusting the cluster growth, and the clustering results are sensitive to these parameter settings. This indicates that the MCODE algorithm is unsuitable for unsupervised learning. CFinder [8] is a local search algorithm that uses the clique percolation technique. CFinder finds the cliques with *k* nodes, called k-cliques, and iteratively merges them if they share (k−1) nodes. Because CFinder must search for cliques to find each cluster, its efficiency and scalability are typically limited, particularly if the network is large and has complex connectivity. The graph entropy (GE) algorithm [9,10] also performs a local search following the seed growth procedure. For each cluster, the selected seed node grows based on the novel metric of GE. Because these local search algorithms find each cluster independently, the resultant clusters can overlap.

Recent studies have emphasized the importance of detecting overlapping clusters. For example, overlapping MCL, which is a method for iteratively forming overlapping clusters [11]; the overlapping cluster generator as a method to use extended modularity for overlapping clusters [12]; pairwise constraint non-negative matrix tri-factorization, which is a method for finding overlapping functional modules based on the matrix [13]; and a method for forming nested clusters with a greedy search algorithm [14] have recently been proposed.

In this study, we verified the role of predicting functional modules from PPI networks using unweighted and weighted GE algorithms. The accuracy of the GE algorithm was compared with that of competitive graph clustering algorithms. We also assessed the contributions of overlapping clusters in terms of functional module prediction.

## 2. Materials and Methods

### 2.1. PPI Datasets

We used two datasets, STRING and BioPlex, as PPI networks for Homo sapiens. The STRING database [15,16] provides broadly integrated interactions and a confidence score for each interaction. The confidence score of STRING [17] corresponds to the probability of finding a linked protein within the same pathway in KEGG [18]. For PPIs from STRING, we used the physical links of their confidence scores limited to 700 or higher. The BioPlex network [19] consists of PPIs obtained using high-throughput affinity-purification mass spectrometry. A unique gene symbol was used for each protein in both datasets and was capitalized. For PPI networks from STRING and BioPlex, 4,338,217 and 118,162 links were used, respectively, by removing redundant links and self-loops.

To analyze the weighted networks, we used the confidence scores of PPIs in STRING as the probabilistic weights of edges. We also applied topological weights to PPIs in STRING and BioPlex by computing the ratios of the common neighboring nodes using the Jaccard index.

### 2.2. References

To measure clustering accuracy on the PPI networks, we compared the clustering results with protein complexes and Gene Ontology (GO) annotation data. Protein complexes were collected from both large- and small-scale experimental results in CORUM [20] and PCDq [21]. The integrated dataset included 2576 distinct proteins.

GO [22] is the most widely referenced ontology database unifying biological representation and provides annotations of molecular products to the biological descriptions based on published evidence. For GO annotations, we combined the terms of the biological process and molecular function sub-ontologies to make 16,588 reference clusters. GOATOOLS [23] was used to identify each description for annotation.

### 2.3. Graph Clustering Algorithms

#### 2.3.1. Graph Entropy Algorithm

A previous study [9,10] introduced a graph clustering algorithm based on the information-theoretic definition of GE which is a measure of modularity in a graph. Suppose an undirected graph is partitioned into *k* subgraphs, $C_{1}$, $C_{2}$, ⋯, $C_{k}$; the entropy of each node *v* is computed as follows:

(1) $$e\left(v\right)=-\sum_{i=1}^{k}p\left(x_{i}\right)\log p\left(x_{i}\right)$$

where $p(x_{i})$ is the ratio of the edges between *v* and the nodes of $C_{i}$ to all edges of *v*. The entropy e(G) of a graph *G* is then defined as the sum of the node entropies for all nodes in *G*.

(2) $$e\left(G\right)=\sum_{i=1}^{N}e\left(v_{i}\right)$$

where *N* is the total number of nodes in *G*. The lowest GE in this equation indicates the highest modularity of the partition of *G*.

The GE algorithm employs the seed–growth procedure, which selects a node as an initial seed cluster and grows the seed cluster to optimize graph modularity. The definition of GE is applied to the local optimization of a cluster. The graph is partitioned into two subgraphs: a seed cluster and the other part. The seed cluster grows to search for the lowest GE. Here, the entropy of each node is computed as follows:

(3) $$e\left(v\right)=-p\left(x_{i}\right)\log p\left(x_{i}\right)-p\left(x_{o}\right)\log p\left(x_{o}\right)$$

where $p(x_{i})$ is the ratio of the edges between *v* and any node inside the seed cluster, and $p(x_{o})$ is the ratio of the edges between *v* and any node outside the seed cluster.

In the GE algorithm, the seed–growth process iterates to find a set of clusters. Because each cluster is generated independently, the resultant clusters can overlap with each other even though a seed node is selected outside the clusters that are found during the preceding iterations. A stepwise description of the GE algorithm is provided below. Similar to the definition of a neighbor of a node *v* as a node linked to *v*, the neighbor of a cluster *C* is defined as a node outside *C* that is linked to any node in *C*.

1. Select a seed node. Among the nodes that are not in the output clusters from Step 6, select the one with the highest degree as the seed node.
2. Form an initial seed cluster including the seed node and its neighbors.
3. Delete each neighbor of the seed node iteratively from the seed cluster if GE decreases. Check the neighbors in descending order of their degrees.
4. Add each neighbor of the seed cluster iteratively into the seed cluster if GE decreases. Check the neighbors in descending order of their degrees.
5. Output the seed cluster if partitioning the graph by the cluster results in the lowest GE.
6. Repeat Steps 1–5 to output a set of clusters until no seed node remains.

#### 2.3.2. Weighted GE Algorithm

The GE algorithm can be applied to a weighted graph. The weight of each edge indicates the strength of the interaction. To detect strongly connected clusters from a weighted graph, the equation for node entropy should be upgraded. In this study, we tested two methods to compute the weighted entropy of each node. The first method weighs the two factors of Equation (3) using the sums of the edge weights.

(4) $$e\left(v\right)=-W_{i}\cdot p\left(x_{i}\right)\log p\left(x_{i}\right)-W_{o}\cdot p\left(x_{o}\right)\log p\left(x_{o}\right).$$

In this equation, $W_{i}=\sum_{i=1}^{m}w_{i}$, where *w* is an edge weight, and *m* is the number of edges of *v* that are linked to the nodes inside the seed cluster. In other words, the weight $W_{i}$ is the sum of the edge weights between *v* and the nodes inside the seed cluster. Similarly, $W_{o}=\sum_{i=1}^{k}w_{i}$, where *k* is the number of edges of *v* that are linked to the nodes outside the seed cluster. The weighted GE algorithm using Equation (4) is referred to as GE with multiplied weights (GE-MW). The second method replaces the ratios of edges in Equation (3) with the weighted ratios.

(5) $$e\left(v\right)=-\frac{W_{i}}{W_{i}+W_{o}}\log\frac{W_{i}}{W_{i}+W_{o}}-\frac{W_{o}}{W_{i}+W_{o}}\log\frac{W_{o}}{W_{i}+W_{o}}.$$

The weighted GE algorithm using Equation (5) is referred to as GE with weighted ratios (GE-WR).

### 2.4. Evaluation of Clustering Accuracy

The metrics of average *F*-score (Equation (6)) and average precision (Equation (7)) were used to evaluate the accuracy of clusters in comparison with protein complexes or GO annotations. The highest value of each cluster set was obtained and the collected values were averaged over all resultant clusters. In this evaluation, we excluded the proteins that do not exist in PPIs from the references. We also excluded the proteins that do not exist in the reference set from the clusters.

Let the clusters be a set $\left\{C_{1},C_{2},\cdots ,C_{l}\right\}$ and the reference be a set $\left\{r_{1},r_{2},\cdots ,r_{p}\right\}$. The precision of a cluster $C_{i}$ compared to $r_{j}$ can be expressed as follows: $P_{ij}=|C_{i}\cap r_{j}|/|C_{i}|$. The recall of a cluster $C_{i}$ compared to $r_{j}$ can be expressed as follows: $R_{ij}=|C_{i}\cap r_{j}|/|r_{j}|$. The average *F*-score $\bar{F}$ and average precision $\bar{P}$ are expressed using Equations (6) and (7), respectively.

(6) $$\begin{array}{ccc} \bar{F} & = & \frac{1}{l}\sum_{i=1}^{l}\left\{\max_{j=1}^{p}\left(2\times \frac{P_{ij}\times R_{ij}}{P_{ij}+R_{ij}}\right),\;i=1,2,\cdots ,l\right\} \end{array}$$

(7) $$\begin{array}{ccc} \bar{P} & = & \frac{1}{l}\sum_{i=1}^{l}\left\{\max_{j=1}^{p}P_{ij},\;i=1,2,\cdots ,l\right\} \end{array}$$

In order to measure the proportion of functionally homogeneous modules in the set of clusters, we referred to a related previous study [24]. Among all clusters, we measured the proportion of the clusters with precision of 0.6 or greater in comparison with GO annotations.

The feasibility of overlapping cluster detection was also evaluated. A node in a PPI network that appears twice or more in the set of clusters is defined as an overlapping node, and a cluster that includes at least one overlapping node is defined as an overlapping cluster. We evaluated the accuracy of the overlapping clusters obtained through each method by comparing them with the references.

## 3. Results

### 3.1. Experimental Settings

We implemented the unweighted and weighted GE algorithms MCODE, CFinder, InfoMap, and MCL for accuracy comparison. Among the selected algorithms, GE and InfoMap do not have any parameters. However, the other methods require parameter settings. We used the same parameter settings for these unsupervised methods on two datasets, STRING and BioPlex. The recommended values from the original study that introduced each method were applied.

MCODE requires many parameter settings. The “degree cutoff” parameter controls the minimum degree of a node to be scored, the “node density cutoff” parameter describes a density threshold for neighbors of a current cluster to be added, the “node score cutoff” parameter controls the score of a node to be added to the current cluster, the “*k*-core” parameter filters out the clusters that do not contain the nodes of at least *k* degrees, and the “max depth” parameter limits the distance from the seed node. Our experimental settings for MCODE parameter values are as follows: we set the degree cutoff to 2, node density cutoff to 0.1, node score cutoff to 0.2, and *k*-core to 2.

CFinder must specify the *k* value to search for *k*-cliques. It also runs in a weighted network with an intensity parameter. A clique is added to a cluster only if its intensity, the geometric average of edge weights in the clique, is larger than a threshold. We used *k* = 3 and intensity threshold = 0 for unweighted networks and intensity threshold = 0.5 for weighted networks.

MCL requires inflation as a parameter that controls the extent of strengthening and weakening. This parameter influences the granularity of clusters. We set inflation = 3. The CDLI [25] and NetworkX [26] libraries were used to implement MCL and InfoMap.

### 3.2. Clustering Results

We excluded singletons having only one node from the obtained clusters. The number of clusters and the average cluster size are compared in Table 1.Comparing the clustering results on the STRING PPI dataset, which is a large network, the GE and MCL algorithms generated a larger number of clusters than the others. This indicates that they become reliable methods in genome-wide analysis of large networks. Conversely, it was confirmed that such a number could not be obtained in the case of a small network, the Bioplex PPI dataset. The unweighted GE algorithm removed a large number of singletons obtained from the small network.

For the reference datasets, the average size of protein complexes was 4.4, and that of GO annotations was 19.9. The clustering results of GE, CFinder, and MCL had an average size similar to those values. However, the clustering results of MCODE and InfoMap had a significantly larger average size than the references.

### 3.3. Accuracy Evaluation of Clusters

The performance of the selected graph clustering algorithms was evaluated by comparing their clustering results with two reference datasets, protein complexes and GO annotations. Table 2 shows $\bar{F}$ scores, and Table 3 shows $\bar{P}$ scores and the proportion of functionally homogeneous modules. For the STRING dataset, the GE, MCODE, InfoMap, and MCL algorithms were applied. Because CFinder is not suitable for application to a large complex network, it could not be tested with the STRING dataset. In our experiment, CFinder was not completed within 50 h under the specifications of Core i9, DDR4 32GB, and RTX 3070. However, for the BioPlex dataset, CFinder and the above four methods were implemented. The elapsed time of CFinder for the BioPlex dataset was 2 h 6 min, whereas GE produced an entire set of clusters in 7 min.

To evaluate the edge weighting, three cases of unweighted, probabilistic, and topological weights were examined. The confidence scores of PPIs in the STRING dataset were used as the probabilistic weights. In summary, for the STRING dataset, all three cases were applied, whereas two cases—unweighted and topological weights—were applied for the BioPlex dataset. To implement weighted GE, we used the GE-WR in Equation (5).

In the $\bar{F}$-score evaluation of Table 2, in the case of the STRING dataset, GE, InfoMap, and MCL excelled in comparison with protein complexes, and in comparison with GO annotations, GE and InfoMap stood out. In the case of BioPlex dataset, GE, CFinder, and MCL excelled in comparison with protein complexes, and GE and MCL excelled in comparison with GO annotations. That is, the GE algorithm took precedence in all four cases applied. The $\bar{P}$ score in Table 3 also showed a similar pattern. It is also noteworthy that the proportion of functionally homogeneous modules among the clusters from the GE algorithm is upstream. This means that most clusters from the GE algorithm are composed of proteins with the same function.

### 3.4. Accuracy Evaluation of Overlapping Clusters

Among the graph clustering algorithms selected for our experiment, the partition-based methods of InfoMap and MCL are unable to detect overlapping clusters. For GE, MCODE, and CFinder, the ratios of overlapping clusters are listed in Table 4. MCODE produced the highest ratio of overlapping clusters. However, the number of clusters of MCODE was significantly smaller than that of GE.

We evaluated the accuracy of the overlapping clusters collected from the clustering results of GE, MCODE, and CFinder. Table 5 shows the $\bar{F}$ scores measured for overlapping clusters only. It can be observed that as the number of clusters is reduced, the overall accuracy decreases. For the STRING dataset, GE using probabilistic weights had the highest $\bar{F}$ score (0.375) compared to protein complexes, and unweighted GE had the highest $\bar{F}$ score (0.537) compared to GO annotations. For the BioPlex dataset, CFinder using topological weights had the highest $\bar{F}$ score (0.432) compared to protein complexes, and GE using topological weights had the highest $\bar{F}$ score (0.359) compared to GO annotations. Overall, in the evaluation of overlapping clusters, GE exhibited the best performance.

We also compared the precision of the overlapping clusters to assess whether the members of an overlapping cluster were included in the same protein complex or GO annotation. As shown in Table 6, the unweighted GE method showed the highest precision for all PPI datasets and references. For the STRING dataset, the average precision was 0.308 compared to protein complexes and 0.880 compared to GO annotations. For the BioPlex dataset, the average precision was 0.575 compared to protein complexes and 0.915 compared to GO annotations. When the overlapping clusters are compared to GO annotations, the $\bar{P}$ scores in Table 6 are remarkably higher than the $\bar{F}$ scores in Table 5. This result was caused by the relatively large size of the GO annotations used as a reference, as well as the large number of GO annotations.

**Figure 1:**
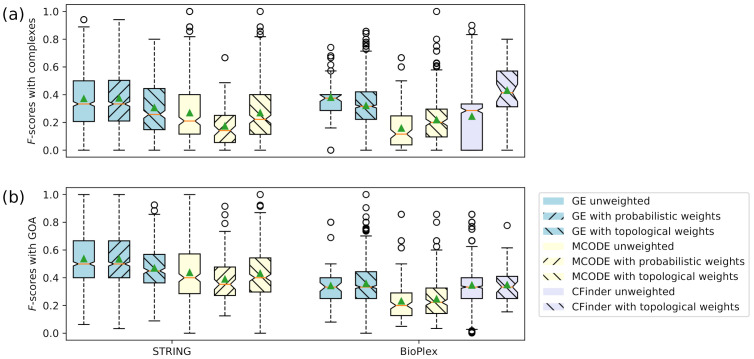
Boxplots of *F*-score distributions of overlapping clusters. (**a**) *F*-scores of overlapping clusters compared to protein complexes. (**b**) *F*-scores of overlapping clusters compared to GO annotations. The green solid triangles and orange lines represent the mean and median values of the distributions, respectively.

**Figure 2:**
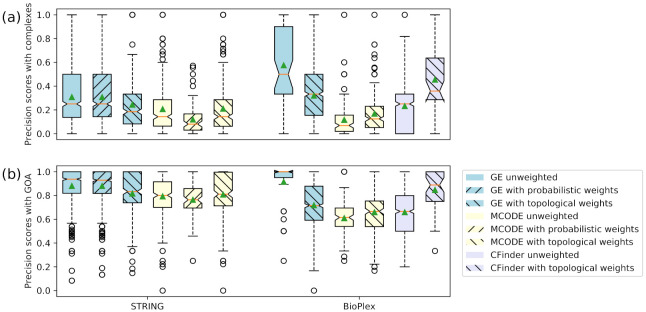
Boxplots of precision distributions of overlapping clusters. (**a**) Precision of overlapping clusters compared to protein complexes. (**b**) Precision of overlapping clusters compared to GO annotations. The green solid triangles and orange lines represent the mean and median values of the distributions, respectively.

For a more detailed comparison of the accuracy of overlapping clusters, Figure 1 and Figure 2 show the distributions of the values from each accuracy metric using boxplots. The distributions of *F*-scores of the overlapping clusters compared to protein complexes and GO annotations (shown as GOA) are displayed in Figure 1. The distributions of precision scores of the overlapping clusters are also examined in Figure 2. In the case of the median and mean values, results similar to those in Table 5 and Table 6 can be confirmed. Overall, the distributions demonstrate that unweighted GE and GE with probabilistic weights have higher accuracy than the other cases.

From the precision comparison in Figure 2, it can be seen that the comparison with GO annotations of Figure 2b shows a higher precision value than the comparison with protein complexes of Figure 2a due to larger and more reference clusters of GO annotations than protein complexes. That is, precision is higher because the average size of the reference clusters of GO annotations (19.9) is significantly larger than that of protein complexes (4.4), and the number of reference clusters of GO annotations (16,588) is greater than the number of protein complexes (2576). Larger and more references give a comparative advantage of higher precision.

### 3.5. Biological Aspects of Clusters from GE Algorithms

To examine the biological aspects of the clustering results from the unweighted and weighted GE algorithms, the STRING dataset was used because of the larger number of PPIs. We considered two aspects: First, the biological suitability of the proteins that appeared most frequently in the clusters was investigated with reference to previous studies, as described in Table 7. Second, novel members of known functional modules from GO were predicted based on the clustering results, as shown in Table 8. Unlike the previous calculation, the *F*-scores compared to GO annotations were obtained without removing the exclusive proteins from the reference. Newly discovered proteins in the clusters with an *F*-score greater than 0.9 were treated as novel members.

Table 6 shows that two high-frequency classes of proteins (Rab1 and ITSN) are involved in the regulation of many other proteins, and the third class of proteins, CaM, is involved in increasing the interaction affinity of many proteins. These functional descriptions explain why overlapping proteins appear so frequently across clusters.

In Table 8, two GO terms with an *F*-score close to 1, that is, GO:0019054 and GO:0070125, were selected. In the case of GO:0019054, its function is described as modulation by virus of host cellular process, and the missing element (KPNA6) can be filled in the Karyopherin proteins; this is easy to reveal intuitively, as confirmed by connections on the PPI network. A recent study [33] also reported that KPNA6 is necessary for replicating viruses such as Zika virus.

In the case of GO:0070125, its function is described as mitochondrial translational elongation, and the newly appeared members were different for each algorithm; therefore, the *F*-score is also different. First, the novel proteins common to all algorithms are AC004556.3, AC139530.2, HDDC3, HIBCH, ICT1, MRRF, MTIF2, MTIF3, MTRF1L, RPL23L, and RPMS17. Among them, ITCN1 has been reported as a putative factor for mitochondrial translational release [34]. A previous study [35] also reported that MTRF1 is a mitochondrial translational release factor, and MRRF is required for ribosome recycling at the termination of mitochondrial translation. Another study [36] reported that MTIF2 and MTIF3 are two initiation factors involved in mitochondrial translation. As mitochondrial ribosomal proteins, RPL23L and RPMS17 are aliases of MRPL23 and MRPS17 from GO annotation, respectively.

Among the novel members of GO:0070125, the exclusive proteins for each method were as follows: using unweighted GE, the three exclusive proteins from GO annotation were MRPL23, MRPL58, and TSFM, and the two exclusive novel proteins are C12ORF65 and MTG2. C12ORF65, also known as mitochondrial translation release factor in rescue (MTRFR), has been reported to prevent aberrant translation during elongation [37]. MTG2, also known as GTPBP5 according to the HGNC symbols, has been reported to be required for mitochondrial translation [38]. Using GE with probabilistic weights, the two exclusive proteins from GO annotation were MRPL23 and MRPL58, and the two exclusive novel proteins were C12ORF65 and MTG2, identical to those determined using unweighted GE. Using GE with topological weights, the three exclusive proteins from GO annotation were MRPL23, MRPL58, and TSFM, and the three exclusive novel proteins were GUF1, PDF, and SOD2. According to the gene nomenclature, GUF1 is known as a translation factor, mitochondrial, or GTP-binding elongation factor; PDF is known as peptide deformylase, mitochondrial; and SOD2 is known as superoxide dismutase 2, mitochondrial.

## 4. Discussion and Conclusions

GE is a novel metric to quantify the modularity of a set of subgraphs (i.e., clusters) in a large, complex network. The GE-based graph clustering algorithm, which iteratively performs a local search to detect an optimal cluster with the lowest GE, was recently proposed. This algorithm can also be extended to the versions for a weighted network, a graph with edge weights. By applying the unweighted and weighted GE algorithms to PPI networks and evaluating their performance, this study confirms their validity for predicting functional modules of proteins.

Unlike other networks, the major property to be considered in a PPI network is modularity. We applied the GE algorithm to a random network with the same number of nodes and edges (in both the Erdos–Renyi method [39] and Knuth method [40]) and found that no clusters were created and only singletons were left. Even in the case of a node with a high degree, most of its neighbors are eliminated during the node removal stage of the GE algorithm because of low modularity. In other words, the connections in random networks cannot be measured because they are literally random, whereas in PPI networks, clusters of proteins can be detected because similar proteins tend to be linked together as protein complexes or functional modules.

Our clustering results have two major implications. First, the GE algorithms are particularly suitable for genome-wide analysis of PPI networks. Their clustering results most closely represent the reference datasets, a set of protein complexes at the genome scale and comprehensive functional modules in GO annotations, in terms of the number of clusters and the average cluster size. Second, the GE algorithm is suitable for predicting functional modules. That is, it belongs to the upper group in a comparison of prediction accuracy and homogeneity of functional modules and has a comparative advantage in accuracy, especially in a comparison of overlapping clusters.

We propose the following two implications from a biological perspective: First, a protein that occurs in many overlapping clusters can be biologically justified based on the functions it performs. Our results confirmed that such proteins are involved in regulation or interaction affinity. A previous study [12] reported that such proteins are involved in regulating and binding activity. It has also been reported that a large number of proteins are involved in the regulation of endocytosis and cell signaling [41]. Second, we propose novel proteins for annotating the GO terms. This may imply the discovery of novel pathways, such as disease–gene associations [42,43].

Finally, the following limitations were identified in this study. The STRING database has an advantage in that it supports a vast amount of interactome data. However, it provides Ensembl protein IDs, whereas gene symbols are commonly used in other datasets. There might be a limit to completely converting Ensembl protein IDs into gene symbols or vice versa. If the aliases for the gene symbols of all proteins are investigated and standardized, better results can be confirmed. There are also cases in which publication bias is inevitable in known PPI networks [44], and attempts to overcome this issue are still insufficient for a genome-wide study [45]. If these limitations are overcome, more accurate and useful results can be expected for research on genome-wide large PPI networks.

## Figures and Tables

**Table 1:** Clustering results of the selected graph clustering algorithm.

PPI Network	Algorithm	Weight	Number of Clusters	Average Cluster Size
STRING	GE	Unweighted	995	19.9
		Probabilistic	982	20.5
		Topological	444	29.9
	MCODE	Unweighted	298	71.2
		Probabilistic	85	172.7
		Topological	296	65.2
	InfoMap	Unweighted	217	44.0
		Probabilistic	219	43.6
		Topological	90	79.3
	MCL	Unweighted	1061	8.9
		Probabilistic	781	11.2
		Topological	478	9.2
BioPlex	GE	Unweighted	126	4.2
		Topological	1188	7.8
	MCODE	Unweighted	113	237.8
		Topological	237	70.5
	CFinder	Unweighted	823	13.1
		Topological	145	10.4
	InfoMap	Unweighted	514	27.2
		Topological	79	109.5
	MCL	Unweighted	3087	3.2
		Topological	154	7.0

**Table 2:** $\bar{F}$ scores of clusters from the selected graph clustering algorithms.

PPI Network	Algorithm	Weight	$\bar{F}$ Score with Protein Complexes	$\bar{F}$ Score with GO Annotations
STRING	GE	Unweighted	0.567	0.554
		Probabilistic	0.572	0.552
		Topological	0.521	0.517
	MCODE	Unweighted	0.465	0.487
		Probabilistic	0.297	0.397
		Topological	0.483	0.494
	InfoMap	Unweighted	0.597	0.587
		Probabilistic	0.570	0.584
		Topological	0.593	0.600
	MCL	Unweighted	0.547	0.495
		Probabilistic	0.538	0.494
		Topological	0.592	0.533
BioPlex	GE	Unweighted	0.506	0.435
		Topological	0.536	0.389
	MCODE	Unweighted	0.316	0.254
		Topological	0.396	0.318
	CFinder	Unweighted	0.483	0.377
		Topological	0.661	0.433
	InfoMap	Unweighted	0.440	0.296
		Topological	0.372	0.388
	MCL	Unweighted	0.534	0.378
		Topological	0.643	0.456

**Table 3:** $\bar{P}$ scores and the proportion of functionally homogeneous modules among clusters from the selected graph clustering algorithms.

PPI Network	Algorithm	Weight	$\bar{P}$ Score with Protein Complexes	$\bar{P}$ Score with GO Annotations	Proportion of Functionally Homogeneous Modules (%)
STRING	GE	Unweighted	0.542	0.902	98.2
		Probabilistic	0.543	0.902	98.2
		Topological	0.482	0.881	97.5
	MCODE	Unweighted	0.432	0.856	94.3
		Probabilistic	0.238	0.806	94.1
		Topological	0.451	0.875	95.6
	InfoMap	Unweighted	0.692	0.932	93.6
		Probabilistic	0.660	0.929	93.7
		Topological	0.580	0.892	95.6
	MCL	Unweighted	0.583	0.895	92.5
		Probabilistic	0.578	0.903	96.4
		Topological	0.619	0.943	97.7
BioPlex	GE	Unweighted	0.775	0.911	93.2
		Topological	0.577	0.791	82.3
	MCODE	Unweighted	0.296	0.690	79.6
		Topological	0.377	0.769	81.0
	CFinder	Unweighted	0.538	0.745	74.5
		Topological	0.714	0.930	97.9
	InfoMap	Unweighted	0.413	0.658	64.1
		Topological	0.356	0.795	86.1
	MCL	Unweighted	0.657	0.754	64.0
		Topological	0.787	0.947	95.8

**Table 4:** Proportion of overlapping clusters from graph clustering algorithms.

PPI Network	Algorithm	Weight	Number of Clusters	Number of Overlapping Clusters	Proportion (%)
STRING	GE	unweighted	995	778	78.1
		probabilistic	982	756	76.9
		topological	444	277	62.3
	MCODE	unweighted	298	250	83.8
		probabilistic	85	80	94.1
		topological	296	230	77.7
BioPlex	GE	unweighted	126	55	43.6
		topological	1188	854	71.8
	MCODE	unweighted	113	112	99.1
		topological	237	192	81.0
	CFinder	unweighted	823	783	95.1
		topological	145	41	28.2

**Table 5:** $\bar{F}$ scores of overlapping clusters from GE, MCODE, and CFinder.

Graph Clustering Algorithm	$\bar{F}$ Score with Protein Complexes	$\bar{F}$ Score with GO Annotations
STRING GE unweighted	0.371	0.537
STRING GE with probabilistic weights	0.375	0.535
STRING GE with topological weights	0.307	0.470
STRING MCODE unweighted	0.269	0.438
STRING MCODE with probabilistic weights	0.174	0.390
STRING MCODE with topological weights	0.270	0.432
BioPlex GE unweighted	0.381	0.342
BioPlex GE with topological weights	0.321	0.359
BioPlex MCODE unweighted	0.159	0.231
BioPlex MCODE with topological weights	0.219	0.248
BioPlex CFinder unweighted	0.244	0.347
BioPlex CFinder with topological weights	0.432	0.349

**Table 6:** $\bar{P}$ scores of overlapping clusters from GE, MCODE, and CFinder.

Graph Clustering Algorithm	$\bar{P}$ Score with Protein Complexes	$\bar{P}$ Score with GO Annotations
STRING GE unweighted	0.308	0.880
STRING GE with probabilistic weights	0.308	0.880
STRING GE with topological weights	0.244	0.817
STRING MCODE unweighted	0.207	0.793
STRING MCODE with probabilistic weights	0.119	0.764
STRING MCODE with topological weights	0.211	0.807
BioPlex GE unweighted	0.575	0.915
BioPlex GE with topological weights	0.319	0.718
BioPlex MCODE unweighted	0.117	0.610
BioPlex MCODE with topological weights	0.168	0.659
BioPlex CFinder unweighted	0.232	0.659
BioPlex CFinder with topological weights	0.455	0.847

**Table 7:** Functions of overlapping proteins with high frequency in the clusters generated by GE.

Overlapping Proteins	Frequency of Appearance			Reported Function
	STRING GE Unweighted	STRING GE with Probabilistic Weights	STRING GE with Topological Weights	
RAB1A	60	60	10	Rab1 proteins regulate vesicular transport [27]. Rab GTPases regulate membrane traffic and are involved in many cell types [28].
RAB1B	60	60	10	
ITSN1	55	54	10	Intersectins (ITSNs) regulate endocytosis and cell signaling [29]. ITSNs may regulate the interactions of various functions [30].
ITSN2	55	54	10	
CALM1	53	51	15	Calmodulin (CaM) is an essential protein for calcium ion sensing and signal transduction [31]. CaM enhances the interaction affinity of many proteins [32].
CALM2	53	51	15	
CALM3	53	51	15	

**Table 8:** Proposing novel proteins for additional annotation to GO terms.

GO Term	GO Name	GO Annotated Proteins	Novel Proteins	Algorithm	*F*-Score
GO:0019054	modulation by virus of host cellular process	KPNA1, KPNA2, KPNA3, KPNA4, KPNA5, KPNA7, KPNB1	**KPNA6**	GE unweighted	0.933
			**KPNA6**	GE with probabilistic weights	0.933
GO:0070125	mitochondrial translational elongation	AURKAIP1, CHCHD1, DAP3, ERAL1, GADD45GIP1, GFM1, GFM2, MRPL1, MRPL10, MRPL11, MRPL12, MRPL13, MRPL14, MRPL15, MRPL16, MRPL17, MRPL18, MRPL19, MRPL2, MRPL20, MRPL21, MRPL22, MRPL23, MRPL24, MRPL27, MRPL28, MRPL3, MRPL30, MRPL32, MRPL33, MRPL34, MRPL35, MRPL36, MRPL37, MRPL38, MRPL39, MRPL4, MRPL40, MRPL41, MRPL42, MRPL43, MRPL44, MRPL45, MRPL46, MRPL47, MRPL48, MRPL49, MRPL50, MRPL51, MRPL52, MRPL53, MRPL54, MRPL55, MRPL57, MRPL58, MRPL9, MRPS10, MRPS11, MRPS12, MRPS14, MRPS15, MRPS16, MRPS17, MRPS18A, MRPS18B, MRPS18C, MRPS2, MRPS21, MRPS22, MRPS23, MRPS24, MRPS25, MRPS26, MRPS27, MRPS28, MRPS30, MRPS31, MRPS33, MRPS34, MRPS35, MRPS36, MRPS5, MRPS6, MRPS7, MRPS9, OXA1L, PTCD3, TSFM, TUFM	**AC004556.3, AC139530.2**, C12ORF65, **HDDC3, HIBCH, ICT1, MRRF**, MTG2, **MTIF2, MTIF3, MTRF1L, RPL23L, RPMS17**	GE unweighted	0.914
			**AC004556.3, AC139530.2**, C12ORF65, **HDDC3, HIBCH, ICT1, MRRF**, MTG2, **MTIF2, MTIF3, MTRF1L, RPL23L, RPMS17**	GE with probabilistic weights	0.920
			**AC004556.3, AC139530.2**, GUF1, **HDDC3, HIBCH, ICT1, MRRF**, **MTIF2, MTIF3, MTRF1L**, PDF, **RPL23L, RPMS17**, SOD2	GE with topological weights	0.910

Bold typefaces indicate a common set of novel proteins from each algorithm.

## Data Availability

STRING, https://string-db.org/cgi/download, accessed on 17 February 2021; BioPlex, https://bioplex.hms.harvard.edu/interactions.php, accessed on 17 February 2021; GO annotations, http://geneontology.org/docs/download-go-annotations/, accessed on 27 February 2021.

## Abbreviations

The following abbreviations are used in this manuscript:

PPI	Protein–Protein Interaction
GE	Graph Entropy
GO	Gene Ontology
$\bar{F}$	Average *F*-Score
$\bar{P}$	Average Precision Score
MCL	Markov Clustering
